# Trends in Antimicrobial Drug Resistance of *Streptococcus pneumoniae* Isolates at Jordan University Hospital (2000–2018)

**DOI:** 10.3390/antibiotics8020041

**Published:** 2019-04-12

**Authors:** Malik Sallam, Jumana Abbadi, Anas Natsheh, Nidaa A. Ababneh, Azmi Mahafzah, Gülşen Özkaya Şahin

**Affiliations:** 1 Department of Pathology, Microbiology and Forensic Medicine, School of Medicine, The University of Jordan, Amman 19328, Jordan; jmb_abbadi@hotmail.com (J.A.); natshehanas@gmail.com (A.N.); mahafzaa@gmail.com (A.M.); 2 Department of Clinical Laboratories and Forensic Medicine, Jordan University Hospital, Amman 22362, Jordan; 3 Department of Translational Medicine, Faculty of Medicine, Lund University, 22362 Malmö, Sweden; gulsen.ozkaya_sahin@med.lu.se; 4 Cell Therapy Center (CTC), The University of Jordan, Amman 19328, Jordan; nidaaanwar@gmail.com; 5 Department of Clinical Microbiology, Laboratory Medicine, Skåne University Hospital, 22362 Lund, Sweden

**Keywords:** *Streptococcus pneumoniae*, meningitis, bacteremia, antibiotic, Middle East, Jordan, resistance, trend

## Abstract

Antimicrobial drug resistance (AMR) in pneumococci complicates the treatment of serious pneumococcal infections. Country-specific AMR patterns can help to establish guidelines for empiric therapy. The aim of the current study was to analyze the distribution of AMR among *Streptococcus pneumoniae* isolates at Jordan University Hospital (JUH) during 2000–2018. Paper-based and electronic clinical data registry records from 2000 to 2018 were retrospectively analyzed to study the AMR among pneumococcal isolates at JUH. Temporal trend analysis was done using two-tailed linear-by-linear test for association. The total number of unique pneumococcal isolates that were identified was 556, of which 544 isolates had antimicrobial susceptibility testing results. The most frequent specimens were eye (*n* = 117, 21.0%), bloodstream (*n* = 93, 16.7%) and sputum (*n* = 81, 14.6%). Invasive infections represented 23.6% of all unique isolates. The overall susceptibility of *S. pneumoniae* isolates during the study period to different antimicrobials was: 100% to vancomycin, 97.7% to ceftriaxone, 97.1% to cefotaxime, 94.9% to chloramphenicol, 89.7% to penicillin, 83.8% to levofloxacin, 67.7% to clindamycin and 52.1% to erythromycin. The prevalence of multi-drug resistance (MDR) was 8.6% (95% confidence interval: 6.4–11.5%). Trend analysis showed an increase in the prevalence of non-susceptibility to erythromycin, clindamycin and levofloxacin (*p* < 0.001). MDR prevalence increased from 1.6% in the first quarter to 14.6% in the fourth quarter (*p* < 0.001). The incidence of invasive infections declined over the study period (*p* < 0.001). The increase in the prevalence of AMR and MDR among pneumococcal isolates in Jordan demands judicious use of antimicrobials and regular surveillance of resistance.

## 1. Introduction

*Streptococcus pneumoniae* is a frequent colonizer of the human upper respiratory tract [1,2]. It is the causative agent for a wide variety of life-threatening infections including pneumonia, meningitis and bacteremia [3,4,5]. In addition, *S. pneumoniae* is a leading cause of otitis, sinusitis and bronchitis, particularly in children [6].

Beta-lactam antibiotics (e.g., penicillin, third-generation cephalosporins) are considered the mainstay of treatment for *S. pneumoniae* infections [7,8]. Macrolides (e.g., erythromycin), fluoroquinolones (e.g., levofloxacin), lincosamides (e.g., clindamycin) and vancomycin can be used for management of beta-lactam non-susceptible pneumococcal isolates and for individuals who cannot tolerate beta-lactams [8].

The emergence of antimicrobial drug resistance (AMR) in *S. pneumoniae* can hinder the management of pneumococcal infections [9]. Multi-drug resistance (MDR) among pneumococci is particularly a serious problem which is defined as resistance of an isolate to three or more classes of antibiotics [10,11,12]. The increasing prevalence of AMR among pneumococci poses serious therapeutic problems, mainly in the management of pneumococcal meningitis [13].

The investigation of AMR trends among pneumococcal isolates can help to understand the role of antibiotic therapy practices in the emergence of resistance and to revise the empiric therapy guidelines for pneumococcal disease at a country and in regional levels [11,14,15,16]. A number of factors have been postulated as the underlying causes behind the emergence of AMR, including: the wide availability of antibiotics as over-the-counter drugs in some countries, unnecessary prescriptions by physicians and non-compliance by the patients [17].

An increasing trend in the prevalence of penicillin-non-susceptible pneumococci have been reported in various regions globally [11,18,19,20]. In Jordan, a few reports indicated a similar pattern of an increasing AMR trend among pneumococcal isolates [21,22,23,24]. However, an updated report from the country is needed as the results of the previous reports are either outdated or focused on specific patient groups (e.g., children), in addition to the issues of small sample sizes and short study periods. Thus, the aim of this project was to study the prevalence and temporal trends of AMR among pneumococcal isolates at Jordan University Hospital (JUH) over a period of 19 years using data on all available isolates.

## 2. Methods

### 2.1. Study Design

This retrospective study was carried out using patient data that were collected from January 2000 to August 2018 at Jordan University Hospital (JUH), an academic teaching hospital in Amman, Jordan. JUH has a bed capacity of 550 and annually serves more than 500,000 patients in the outpatient clinics as of January 2019. Manual search (January 2000 till December 2008) and electronic search (January 2009 till August 2018) in the clinical data registry records were conducted by three authors (MS, JA and AN).

### 2.2. Ethical Permission

The study was approved by the Jordan University Hospital ethical review board (IRB/262/2018) in accordance with the declaration of Helsinki.

### 2.3. Identification of S. pneumoniae Isolates and Antimicrobial Susceptibility Testing (AST)

The presumptive identification of *S. pneumoniae* isolates at JUH depends on colonial morphology, alpha-hemolysis on 5% sheep blood agar (SBA) and sensitivity to optochin (5 µg) following a 24-hour incubation in 5% CO_2_. Antimicrobial susceptibility testing (AST) was performed using Kirby-Bauer disk diffusion method. AST results were reported for the following antimicrobials: vancomycin, erythromycin, clindamycin, chloramphenicol ceftriaxone, cefotaxime and levofloxacin. Zone sizes were measured and interpreted in accordance with Clinical & Laboratory Standards Institute (CLSI) guidelines at time of specimen collection. For penicillin, AST was performed by determining the minimum inhibitory concentration (MIC) using the E-test penicillin G strips graduated from 0.016 to 256 μg/mL (BioMérieux). Cerebrospinal fluid (CSF) isolates with MIC ≤ 0.06 µg/mL were considered susceptible to penicillin and those with MIC > 0.06 µg/mL were considered non-susceptible. Non-CSF isolates were classified as susceptible, if the MIC was ≤2.0 µg/mL and non-susceptible if the MIC was >2.0 µg/mL [25,26]. Starting from September 2017 onwards, the identification of pneumococcal isolates and AST were done using Vitek 2 (BioMérieux) automated system. For all methods, CLSI-recommended quality control checks were done on regular basis.

### 2.4. Study Population

Data on patients and *Streptococcus pneumoniae* isolates were collected and included the following: age, gender, type of specimen, specimen request date, AST results (susceptible versus non-susceptible) and MIC for penicillin. Multiple isolates collected from the same patient and on the same day and having the same AST results were considered as a single “unique isolate”. Unique isolates collected from multiple sites were referred to as “multiple”. For isolates that were collected from the same individual on different dates, but having identical AST results, we used the data of the earliest specimen. Isolates from the same individual with different AST results were considered as different unique isolates. Multi-drug resistance (MDR) was defined as non-susceptibility to at least three antimicrobials from the following classes: beta-lactams (penicillin, ceftriaxone or cefotaxime), macrolides (erythromycin), lincosamides (clindamycin) or fluoroquinolones (levofloxacin) [10].

The isolates that were cultured from bloodstream, CSF, peritoneal fluid, pleural fluid or joint fluid were considered as invasive infections, whereas the rest of isolates that were cultured from non-sterile sites were considered non-invasive.

### 2.5. Statistical Analysis

Statistical analysis was conducted through IBM SPSS Statistics 22.0 for Windows. Two-sided Fisher’s exact test (FET) was used when appropriate. Statistical significance was considered for *p* < 0.050. Temporal trends were analyzed using two-sided linear-by-linear test for association (LBL). Trend analyses were conducted by dividing the study period into quarters, each of which represented 56 months. The 95% confidence interval (CI) of the prevalence (Wilson score interval, binomial distribution) was calculated using EpiTools epidemiological calculator available online (http://epitools.ausvet.com.au).

## 3. Results

### 3.1. Characteristics of Streptococcus pneumoniae Isolates at JUH

A total of 596 *S. pneumoniae* isolates were collected at JUH between January 2000 and August 2018 (19 years). Of these isolates, 556 (93.3%) were unique (i.e., collected from different individuals, or collected from the same individual at different time points with different AST profiles). These unique isolates were collected from 534 different individuals (a single isolate from 515 individuals each, two isolates from 17 individuals, three isolates from a single individual and four isolates from a single individual).

**Figure 1:**
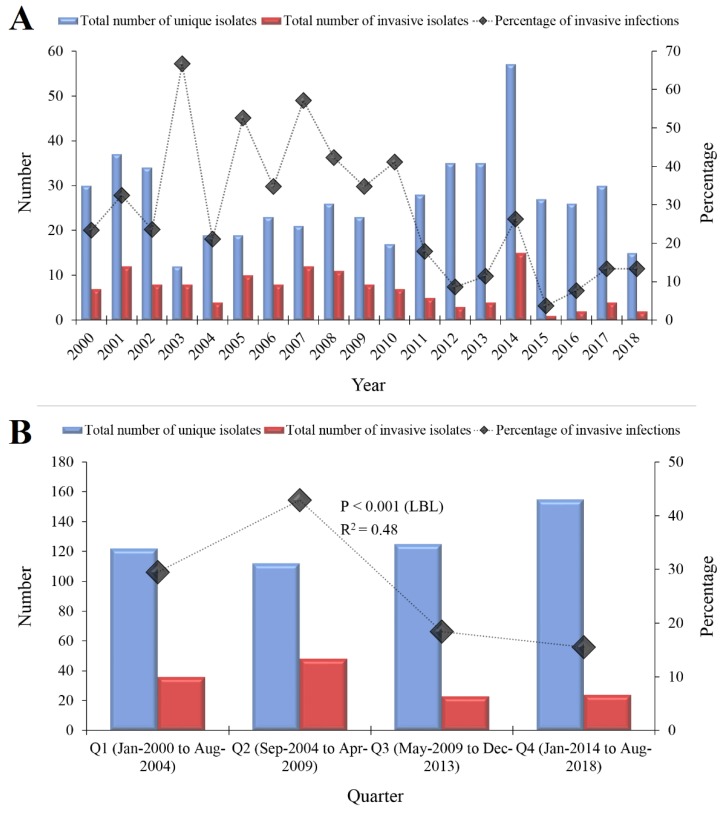
The total number of *Streptococcus pneumoniae* isolates that were detected at Jordan University Hospital during 2000–2018. (**A**) The total number of *S. pneumoniae* isolates stratified per year of isolation are shown in blue and the total number of invasive isolates (isolated from sterile specimens) are shown in red. The line plotted on the secondary axis represents the percentage of invasive isolates per year. (**B**) The total number of *S. pneumoniae* isolates stratified per quarter (each of which represented 56 months) are shown in blue and the total number of invasive isolates are shown in red. The line plotted on the secondary axis represents the percentage of invasive isolates per quarter. A decrease in the proportion of invasive isolates was found over the study period (*p* < 0.001, linear-by-linear (LBL) test for association LBL). R^2^ represent the correlation coefficient of the trend line. The total number of isolates plotted in the figure was 514, as we were not able to retrieve data on specimen type for a total of 42 isolates.

Three-fourths of the total isolates (*n* = 417, 75.0%) were cultured from five sites as follows: eye (*n* = 117, 21.0%), bloodstream (*n* = 93, 16.7%), sputum (*n* = 81, 14.6%), ear (*n* = 76, 13.7%) and nasopharynx (*n* = 50, 9.0%). Data on specimen type could not be retrieved for 42 individuals (7.6%); these isolates were neither CSF nor bloodstream isolates, as CSF and bloodstream specimens are always linked to a specific hospital code. A total of 12 unique isolates in multiple specimens from the same individual were found (2.2%) as follows: seven from CSF and bloodstream, and five from bloodstream and other specimens (peritoneal fluid = 2, plural fluid = 1, joint = 1 and sputum = 1). A total of 131 isolates (23.6%) were obtained from invasive infections (bloodstream = 93, CSF = 17, bloodstream and other sites = 12, peritoneal fluid = 4, joint fluid = 3 and pleural fluid = 2; Table 1). A decrease in the prevalence of invasive isolates was found over the study period from 29.5% in the first quarter to 15.5% in the fourth quarter (*p* < 0.001; LBL, Figure 1).

### 3.2. Characteristics of the Study Population

The total number of unique individuals with pneumococcal isolates was 534. 300 individuals were males (56.2%), and the median age of the individuals at time of specimen collection was 10 years (mean: 23, mode: 1, interquartile range [IQR]: 1–43, range: 1–98, data on age were missing for 33 individuals). Nearly two-thirds of the individuals (*n* = 332) were either ≤10 years or ≥65 years. Characteristics of the study population are summarized in (Table 1).

### 3.3. Antimicrobial Drug resistance Among pneumococcal Isolates in Jordan

The AST reports were found for at least one antibiotic for a total of 544 unique isolates. The overall susceptibility of *S. pneumoniae* isolates during the study period to different antimicrobials was as follows: 100% to vancomycin, 97.7% to ceftriaxone (95% CI: 95.5–98.8%), 97.1% to cefotaxime (95% CI: 94.7–98.4%), 94.9% to chloramphenicol (95% CI: 92.7–96.5%), 89.7% to penicillin (95% CI: 86.3–92.3%), 83.8% to levofloxacin (95% CI: 79.7–87.2%), 67.7% to clindamycin (95% CI: 63.6–71.6%) and 52.1% to erythromycin (95% CI: 47.9–56.3%). The prevalence of MDR was 8.6% (95% CI: 6.4–11.5%). Details of AMR among pneumococcal isolates in the study are summarized in (Table 2).

### 3.4. Variables that were Associated with Higher Prevalence of AMR

Upon investigating the variables that could have a possible association with higher prevalence of AMR, we found that resistance to cefotaxime was higher in males than in females (4.5% versus 0.7%, *p* = 0.049; FET). Cefotaxime resistance was also higher among isolates from invasive infections than non-invasive isolates (8.2% versus 1.2%, *p* = 0.003; FET). In addition, isolates from invasive infections had higher prevalence of resistance to penicillin compared to non-invasive isolates (18.0% versus 7.8%, *p* = 0.007; FET). Conversely, non-invasive isolates had higher prevalence of resistance to clindamycin (35.6% versus 21.7%, *p* = 0.003; FET), to erythromycin (51.0% versus 38.0%, *p* = 0.011; FET) and to chloramphenicol (6.2% versus 1.6%, *p* = 0.037; FET) in comparison to invasive isolates. All other comparisons yielded statistically non-significant differences. Among the CSF or CSF/bloodstream isolates that had AST results (*n* = 19), the prevalence of non-susceptibility to penicillin was 84.2% (95% CI: 62.4–94.5%).

### 3.5. Temporal Changes of AMR Among pneumococcal Isolates in Jordan

**Figure 2:**
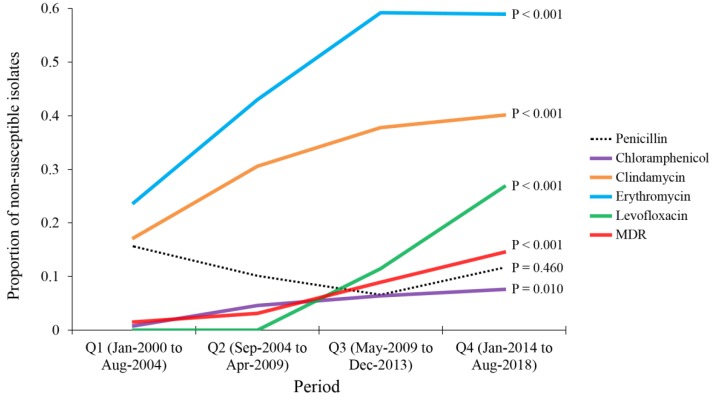
Temporal changes of antimicrobial drug resistance among *Streptococcus pneumoniae* isolates that were detected at Jordan University Hospital during 2000-2018. Analysis was done by dividing the study period into quarters (Q), each of which represented 56 months: Q1→January 2000–August 2004, Q2→September 2004–April 2009, Q3→May 2009–December 2013 and Q4→January 2014–August 2018. *p* values were calculated using linear-by-linear test for association. MDR: multi-drug resistance.

To investigate the temporal trend of AMR prevalence among the unique pneumococcal isolates, we divided the study period into quarters (Q), each of which represented 56 months: Q1→January 2000–August 2004, Q2→September 2004–April 2009, Q3→May 2009–December 2013 and Q4→January 2014–August 2018. We found an increase in the prevalence of non-susceptibility to clindamycin (from 17.2% in Q1 to 40.1% in Q4, *p* < 0.001; LBL), an increase in the prevalence of non-susceptibility to erythromycin (from 23.8% in Q1 to 58.9% in Q4, *p* < 0.001; LBL), an increase in the prevalence of non-susceptibility to levofloxacin (from 0% in Q1 to 26.8% in Q4, *p* < 0.001; LBL) and an increase in the prevalence of non-susceptibility to chloramphenicol (from 0.8% in Q1 to 7.6% in Q4, *p* = 0.010; LBL, Figure 2). In addition, we found an increase in the prevalence MDR from 1.6% in Q1 to 14.6% in Q4 (*p* < 0.001; LBL, Figure 2). For penicillin, no significant change was found in the prevalence of non-susceptibility among pneumococcal isolates during the study period (*p* = 0.460; LBL). However, we found an incremental increase in the median MIC values over the study period (0.38 µg/mL in Q1, 0.50 µg/mL in Q2 and 1.0 µg/mL in Q3 and Q4).

## 4. Discussion

The current study represents a large-scale surveillance update on AMR patterns and trends among *S. pneumoniae* isolates in Jordan over a 19-year period. The study design was based on our attempt to track the long-term changes in the status of antimicrobial drug resistance among pneumococcal isolates in Jordan. Our aim was to assess the changes in resistance patterns that could reflect the local antibiotic prescription practices and treatment guidelines.

In Jordan, pneumococcal conjugate vaccines (PCVs) are not included in the national vaccination schedule, which is related to cost issues (Ministry of Health, Amman, Jordan). However, PCVs have been available since more than a decade for individuals who can afford it. This might explain the significant decrease in the number and proportion of invasive infections over the study period. However, pneumococcal infections in the country continue to represent a huge burden. A recent study from Jordan reported the impact of implementing PCVs and its effect on reducing the carriage rate and AMR among pneumococcal isolates in the individual groups that were studied (infants and children) [27]. Thus, widespread use of PCVs should be considered in Jordan as there is some evidence in literature pointing to possibility of reduction in non-susceptibility to antibiotics following vaccine introduction, even though other study pointed to an opposite effect [28,29].

The main finding of the study was the observation of an increasing prevalence of AMR to drugs that are frequently considered the treatment of choice for pneumococcal diseases [9]. This upward trend in resistance was observed for erythromycin, clindamycin, levofloxacin and chloramphenicol among *S. pneumoniae* in Jordan over the past 19 years. Similar trend was also reported in past and recent studies from other countries and regions worldwide [18,30,31,32]. An interesting finding was related to the pattern of levofloxacin non-susceptibility which was non-existent in the first half of the study and reached a rate of 23.5% in the later half. This finding could be explained by the increased prescription of levofloxacin in the management of pneumococcal disease in Jordan [33]. The emergence of levofloxacin non-susceptibility has been reported in Asia and North America from the early 2000s; however, our finding is among the highest reported rates in the world to the best of our knowledge [33,34,35,36,37].

In addition, the proportion of MDR isolates significantly escalated over the study period. Similar pattern was also reported in a previous study from Jordan [24]. However, our study has the advantage of inclusion of a larger sample size from individuals of different age groups with both invasive and non-invasive isolates. This alarming observation of an increasing prevalence of MDR is in line with other reports globally, particularly from the Asian countries [35,38,39]. The observation of an increased prevalence of AMR and MDR necessitates intervention measures, including ongoing AMR surveillance, antibiotic stewardship, consideration of newer drugs with activity against MDR pneumococci and the consideration of introduction of PCVs which might help to reduce resistance [40,41].

Another finding of the study was the stable trend of non-susceptibility to penicillin among *S. pneumoniae* isolates in Jordan. The overall prevalence of non-susceptibility to penicillin in Jordan during the study period appeared to be low at 10.3%. This finding contrasts the results of the previous and recent reports from the Middle East and North Africa (MENA) region and at the country level [18,21,22,42,43]. The accuracy of this finding is related to our adoption of the latest CLSI guidelines for interpretation of the MIC for pneumococcal isolates [25]. The rationale behind using the revised CLSI breakpoints retrospectively for non-CSF isolates is that it gives a better correlation for in vivo susceptibility and a more accurate perspective in epidemiologic surveillance studies [26]. In spite of finding a stable trend of non-susceptibility to penicillin among pneumococcal isolates in this study, two points should be further clarified. First, the study isolates associated with invasive infections showed significantly higher level of resistance to penicillin (particularly for CSF isolates), which discourage the use of penicillin for empiric therapy of such infections. The other point is the incremental increase in the median MIC for penicillin among all pneumococcal isolates in the study. Taken together, the continuous surveillance of penicillin resistance among pneumococci is strongly recommended to revise the local pneumococcal disease treatment guidelines. Contemporary low levels of penicillin non-susceptibility among non-invasive infections together with high levels of resistance among invasive infections were also reported in recent studies from Palestine and Algeria [44,45].

Despite its low prevalence, the alarming observation of non-susceptibility to third-generation cephalosporins (ceftriaxone and cefotaxime), should be taken into consideration particularly among the invasive isolates. A recent study from Taiwan reported a similar finding and suggested treatment options including penicillin, fluoroquinolones or vancomycin to improve the outcome among the patients infected with ceftriaxone-resistant pneumococci [46].

Several limitation points in the study need to be addressed. First, the lack of serotyping and genotyping results, which precluded the assessment of the pneumococcal strains circulating in Jordan. Second, study subjects that were colonized by pneumococci but without any clinical evidence of infection could not be differentiated due to lack of such clinical data. Third, data on community-acquired versus nosocomial infections were missing. Fourth, the interpretation of AST results was based on visual inspection and zone size measurement before September 2017. Thus, a slight difference in AST results might be expected if reporting was done using MICs. Fifth, multiple unique isolates from the same individual can be representative of a single isolate with AMR evolving over time or due to acquisition of a novel pneumococcal strain. Sixth, we cannot rule out sampling bias, based on the findings of frequent isolates from eye specimens and relatively low number of invasive isolates in our study. All these limitations should be considered in the design of future studies tackling the issue of pneumococcal epidemiology in the country.

## 5. Conclusions

The results of the study can be helpful for the improvement of guidelines for empiric therapy of pneumococcal disease in Jordan. The use of penicillin is strongly discouraged in the management of meningitis locally. Ongoing surveillance of pneumococcal AMR and MDR is strongly recommended together with consideration of implementing PCVs in the country. A follow-up prospective nationwide study is needed to assess the molecular epidemiology of *S. pneumoniae* in Jordan using DNA-based methods.

## Figures and Tables

**Table 1:** Characteristics of the study population and unique *Streptococcus pneumoniae* isolates that were detected at Jordan University Hospital during 2000–2018.

Characteristic	All	
	N ^1^	%
**Gender ^2^**		
Male	300	56.2
Female	234	43.8
**Specimen ^3^**		
Eye	117	21.0
Bloodstream	93	16.7
Sputum	81	14.6
Ear	76	13.7
Nasal	50	9.0
Unknown (non-Blood, non-CSF) ^4^	42	7.6
CSF	17	3.1
Pus	15	2.7
Blood/CSF ^5^	7	1.3
Blood/others ^6^	5	0.9
Others ^7^	53	9.5

^1^ N: Number. ^2^ Gender: for unique individuals. ^3^ Specimen: for unique isolates, hence, the total number was higher than the total number of unique individuals. ^4^ Unknown: information on specimen type could not be retrieved; however, it was possible to confirm that these samples are neither bloodstream nor cerebrospinal fluid (CSF), as they were not assigned with codes of these specimens, ^5^ Blood/CSF: dual identical isolates from the same unique individual were collected from both the bloodstream and CSF. ^6^ Blood/others: dual identical isolates were collected from the same unique individual with isolates collected from bloodstream and non-CSF specimens. ^7^ Others: specimens included: bronchoalveolar lavage fluid (*n* = 11, 2.0%), throat (*n* = 10, 1.8%), sinus aspirate (*n* = 9, 1.6%), urine (*n* = 7, 1.3%), skin (*n* = 5, 0.9%), peritoneal fluid (*n* = 4, 0.7%), joint fluid (*n* = 3, 0.5%), vaginal swab (*n* = 2, 0.4%) and pleural fluid (*n* = 2, 0.4%).

**Table 2:** Antimicrobial susceptibility testing (AST) results among unique *Streptococcus pneumoniae* isolates at Jordan University Hospital (2000–2018).

Antimicrobial ^1^	PG		CRO		CTX		CPL		LVX		CLI		ERY		MDR ^2^	
Characteristic	S ^3^	NS ^4^	S	NS	S	NS	S	NS	S	NS	S	NS	S	NS	YES	NO
**Gender**																
Male	212	26	192	7	189	9	285	19	169	32	198	105	157	147	20	244
Female	152	16	142	1	143	1	219	8	136	27	163	67	123	110	20	182
**Specimen ^5^**																
Invasive	82	18	83	3	78	7	127	2	64	6	101	28	80	49	7	108
Non-invasive	282	24	251	5	254	3	377	25	241	53	260	144	200	208	33	318
**Age group ^6^**																
<11, >64	238	24	217	5	214	7	315	17	208	39	216	116	161	173	27	271
11-64	107	14	102	3	103	3	158	10	92	20	121	49	98	74	12	131
**Period ^7^**																
Q1	54	10	30	0	29	1	121	1	1	0	101	21	93	29	1	63
Q2	62	7	62	6	61	7	103	5	53	0	75	33	61	46	3	91
Q3	127	9	126	1	126	1	146	10	139	18	97	59	64	93	14	143
Q4	121	16	116	1	116	1	134	11	112	41	88	59	62	89	22	129

^1^ Antimicrobial: PG: penicillin, CRO: ceftriaxone, CTX: cefotaxime, CPL: chloramphenicol, LVX: levofloxacin, CLI: clindamycin, ERY: erythromycin. All isolates in our study were susceptible to vancomycin. The total number will not add up to 534 for each antimicrobial as AST was not performed for all antimicrobials for all isolates. ^2^ MDR: Multi-drug resistance. ^3^ S: Susceptible. ^4^ NS: Non-susceptible. ^5^ Specimen: Invasive specimens include those collected from sterile sites: cerebrospinal fluid, bloodstream, pleural, peritoneal and joint fluid. ^6^ Age group: two categories including those younger than 11 years and older than 64 years which was considered a group with higher risk and those between 11 and 64 years as a group with lower risk. Some unique individuals lacked data on age; thus, the total number in this category will be lower than the total number for other categories (gender, specimen and quarter for each antimicrobial). ^7^ Period: the study period was divided into four quarters (Q), each of which represents 56 months as follows: Q1→January 2000–August 2004, Q2→September 2004–April 2009, Q3→May 2009–December 2013 and Q4→January 2014–August 2018.
